# Modelling transitions between egalitarian, dynamic leader and absolutist power structures

**DOI:** 10.1371/journal.pone.0263665

**Published:** 2022-02-14

**Authors:** John Bryden, Eric Silverman, Simon T. Powers

**Affiliations:** 1 School of Biological Sciences, Royal Holloway, University of London, Egham, United Kingdom; 2 Observatory on Socia Media, Indiana University, Bloomington, Indiana, United States of America; 3 MRC/CSO Social and Public Health Sciences Unit, University of Glasgow, Glasgow, United Kingdom; 4 School of Computing, Edinburgh Napier University, Edinburgh, United Kingdom; University of Electronic Science and Technology of China, CHINA

## Abstract

Human groups show a variety of leadership dynamics ranging from egalitarian groups with no leader, to groups with changing leaders, to absolutist groups with a single long-term leader. Here, we model transitions between these different phases of leadership dynamics, investigating the role of inequalities in relationships between individuals. Our results demonstrate a novel *riches-to-rags* class of leadership dynamics where a leader can be replaced by a new individual. We note that the transition between the three different phases of leadership dynamics resembles transitions in leadership dynamics during the Neolithic period of human history. We argue how technological developments, such as food storage and/or weapons which allow one individual to control large quantities of resources, would mean that relationships became more unequal. In general terms, we provide a model of how individual relationships can affect leadership dynamics and structures.

## 1 Introduction

There are many different types of leadership dynamics found in human societies. For instance, many small groups, such as private companies, have a permanent leader. In other groups the leader changes over time, such as in university departments or social societies. These patterns are also seen at larger scales. During the decades before and after Julius Caesar crossed the Rubicon in 49BCE, the Roman Republic transitioned from a system of Consuls who held power for only one year, to the Roman Empire where there was a single Imperator Caesar who ruled for life and passed the title to a chosen successor.

A notable feature of many human groups is that they often do not explicitly coerce their members to join a hierarchy. Instead, soft power and prestige play a strong role [1, 2], with status being an abstraction of more tangible material resources such as land, food, weapons or other commodities [3]. Status is then voluntarily conferred upon leaders by their allies [2–9], with many of these relationships being asymmetric [2, 5, 9–11]. The member with the highest status is usually deemed the leader [2, 4, 6, 8], creating hierarchical societies. Questions remain as to which factors determine why some groups have no leader, others have transient leaders and yet others have relatively permanent leaders.

Considering society at a large scale, we observe a shift between different forms of leadership dynamics in evidence from the Neolithic Era. Before this era, human societies consisted of egalitarian hunter-gatherer groups where material resources such as food were shared relatively equally [12] and leadership roles were facultative and of a temporary duration. There followed a transition to sedentary groups where high-status individuals had more resources but leaders still changed relatively regularly [13]. Finally, hereditary leadership became institutionalised, where the role of a chief was passed down a paternal line, which monopolised most of the resources [14].

Previous work has argued that these shifts were due to social and technological developments, which meant that interactions between individuals became increasingly asymmetric. These asymmetries were likely due to control of agricultural surpluses [15–18], land [15], ideologies [19], or military units and weapons [14]. In light of this evidence, the model we present here investigates how asymmetry in status interaction can generate the different classes of leadership dynamics observed during the Neolithic Era.

Network analysis has proved to be a useful approach for studying the interactions of members of a population [3, 7, 20]. Quantitative study of hierarchical networks is usually static in nature and networks are presented as snapshots in time [21, 22]. However, when using the nodes of a network to represent individuals, the properties of the nodes are often in flux, and the connections between nodes change over time. When there is a feedback effect between node properties and node-to-node connections, the network is said to be coevolutionary. These coevolutionary networks can generate complex dynamics [23, 24].

In order to investigate the factors underlying different types of leadership dynamics, we present a dynamic coevolutionary network which incorporates the *status* of individuals as properties of the nodes. We take status to represent the control of tangible and intangible resources such as food, land, money or other assets, or authority. An edge on the network represents a *relationship* between two individuals, over which exchanges of status are made. An exchange of status might be the trade of goods or services, an employment contract, or a political endorsement. An important factor of our model is the concept that many of the trades in a relationship are somewhat unequal, both in the absolute value assigned to each partner, and the relative value to each partner. Based on this we also specify rules for how edges between nodes are rewired so that individuals can maximise the status they receive. Given these rules, we allow the network to evolve over time and observe its dynamics.

## 2 Model

The model consists of a dynamic network of *n* nodes which represent individual people. All individuals are considered to be identical and are unable to coerce one another to form relationships. Leadership among individuals is solely determined by status. Each individual in the model has a status level which depends on their relationships with others, meaning that status is adjusted according to the status of those who they are linked to. Individuals distribute a proportion of their status amongst those they are linked with, and may not expect the same quantity of status in return. Individuals can change who they associate with according to the marginal utility of the relationships.

Each individual’s node *i* maintains a status *s*_*i*_, which translates to how much influence they have within the group. Status acts as a multivariate aggregate of an individual’s level of money, prestige (titles, jobs, etc), and ownership (land, valuable resources, etc). For simplicity, we assume that individuals must maintain a fixed number of necessary relationships, which is constant for all individuals. These relationships might be needed to participate in society, providing land to live on or food to survive the winter. In our model, each node is assigned λ unidirectional *outgoing* edges which represent their relationships and are linked to other nodes. Nodes can have any number of *incoming* edges from others in the network.

The statuses of the nodes are updated according to their edges in the status update stage, and nodes may rewire an edge in the rewiring stage. The model is run forward in time to observe the distribution of status and changes of that distribution amongst the nodes. We concentrate on looking for *leader* individuals with nodes of high status and check to see whether they were superseded by other leaders. Models are run until patterns of leadership dynamics stabilised, or for a substantially long time (up to 5 million time steps) to confirm that there is an extremely low likelihood of a new leader rising to high status.

### 2.1 Status update stage

**Fig 1:**
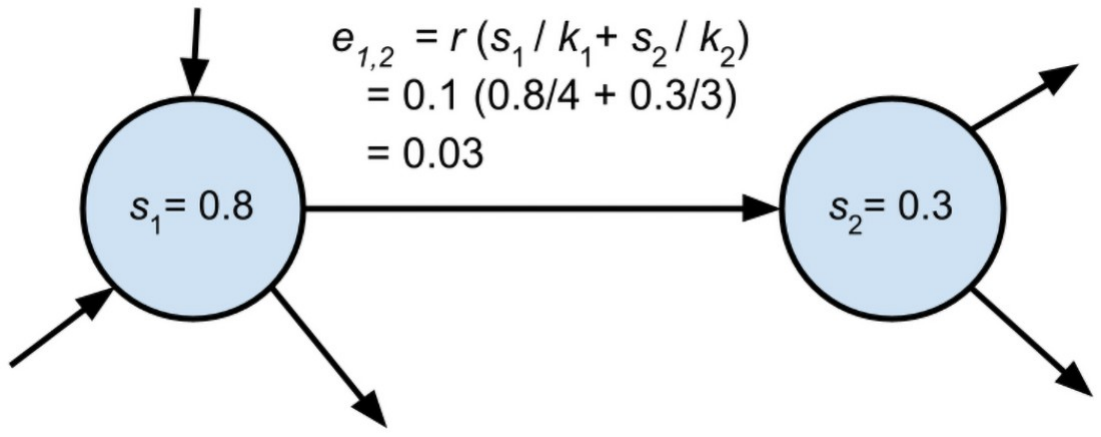
Example of how the status value of edges is calculated. In this example, *s*_1_ will receive 0.03(1 − *q*) status from the edge and *s*_2_ will receive 0.03*q* status.

In the model, a proportion of *r* of each node’s status is distributed amongst each of its edges (including both incoming and outgoing edges). This formalisation of sharing status amongst edges is based on Katz’s prestige measure [25]. For each edge, we assign a temporary status value. This is calculated by adding the status contributions from both of the nodes that are linked. To model unequal relationships we introduce the *inequality parameter* (*q*) which unequally reassigns the edge’s status back to those joined by the edge. In this formulation, the total amount of status in the model is constant. The steps are done in the following order at time *t*:

1. Each unidirectional edge (*i* → *j*) is assigned a temporary status value: *e_i→j_*(*t*) = *rs_i_*(*t*)/*k_i_* + *rs_j_*(*t*)/*k_j_*, where *k*_*i*_ is the degree of node *i* including both incoming and outgoing edges. For an example, see Fig 1.
2. Each node deducts the status distributed to its edges: *s*_*i*_(*t* + 1) = (1 − *r*)*s*_*i*_(*t*).
3. The status of each edge is redistributed back to the nodes: ∀*e_i→j_*, *s_j_*(*t* + 1) = *s_j_*(*t* + 1) + *qe_i→j_* and *s_i_*(*t* + 1) = *s_i_*(*t* + 1) + (1 − *q*)*e_i→j_*

### 2.2 Rewiring phase

In order to maximise status, each individual determines which of its outgoing relationships is of the least value, and, with probability *w* chooses a new relationship according to the following rules.

1. For node *i* at time *t* we identify the edge of minimum value (*i* → j*) from the node’s outgoing edges (*i* → *j*), such that e_i→j_*(*t*) = *min*[*e_i→j_*(*t*)], ∀*j*.
2. With probability *w*, we rewire the edge to a new node by choosing a random node *z* such that there is no edge (*i* → *z*). Delete edge (*i*→j*) and add edge (*i* → *z*).
3. With probability (1 − *w*), we do nothing.

## 3 Results

**Fig 2:**
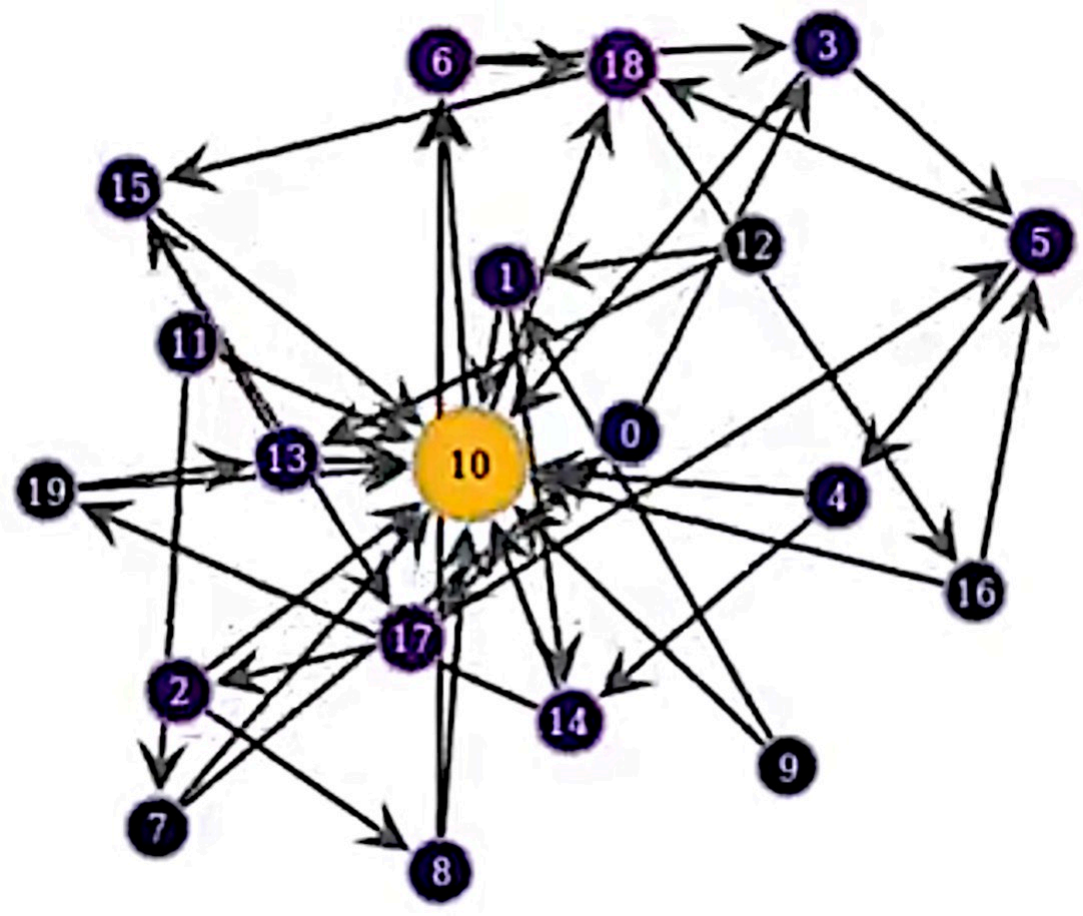
A plot of the network showing a single node with a high level of status compared to the others. Nodes with higher status are lighter coloured and have larger circles. Nodes are marked with ID numbers.

We will present our analysis of the dynamics that result from the interplay between the processes we have defined. Simulations were run of the model choosing parameter values to explore their effects on dynamics over the extremes of their ranges. Depending on the parameters, we either observe relatively equal statuses among the population, or a relatively high status level for one or a few individuals’ nodes. An example of a typical network with a single dominant individual can be seen in Fig 2.

### 3.1 Inequality in relationships affects leadership dynamics

**Fig 3:**
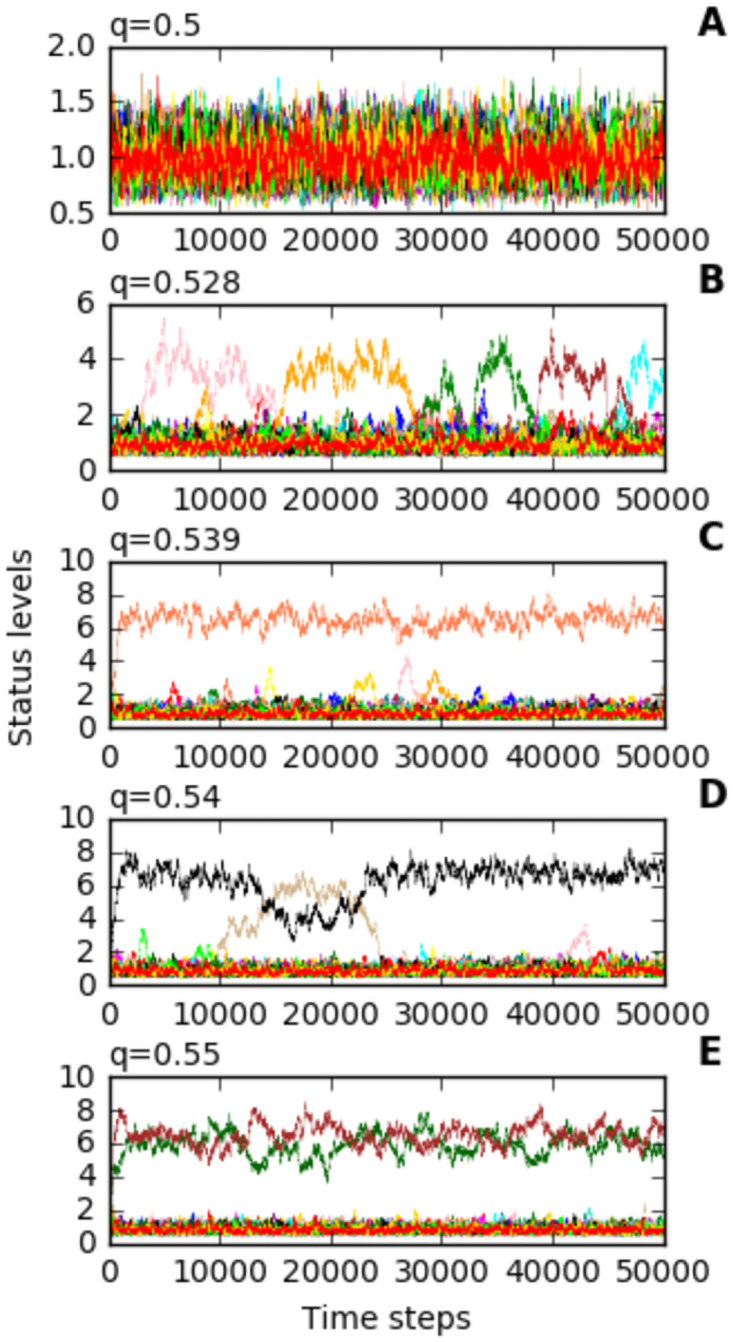
Increasing the inequality parameter takes the model through five different phases. First there is effectively no leader at all (panel A). Then we see that a single individual can rise to a high leadership status, but this is transient and leaders are replaced by other individuals (panel B). The length of time that individuals stay as leader then increases as we increase q until the leader is effectively permanently in charge (panel C). In the next phase, a second individual can rise to a high status alongside the first leader, but these individuals’ leadership position is transient (panel D). Finally, two individuals share leadership status and remain so permanently (panel E). The value of *q* is shown, other parameters are *r* = 0.2, *n* = 50, λ = 3, *w* = 0.5.

A key parameter in the model is the inequality parameter (*q*), which models an unequal transfer of status from a relationship originator to the receiver. As we increase *q*, we observe different phases of dynamics in the model, which are shown in Fig 3. We dub the individual whose node has the highest level of status as the leader. The model exhibits three different types of leadership dynamics: No leader, transient leader(s), and permanent leader(s).

### 3.2 Exploration of a broader range of parameters

We find our simulations demonstrate all three phases of leadership dynamics over a wide range of parameters, including the population size, and numbers of edges. To show this, we run simulation models for each parameter set and record the number of times over the simulation there is a change of individual with the highest status. We find a similar pattern across the parameters tested (see S1–S9 Figs) to that shown in Fig 3. At lower values of *q* there is a very fast turnover of the highest-status individual. As *q* is increased, we find a transient phase where new leaders emerge, but there is still turnover of leaders. At higher values of *q* there are very few new leaders. When leaders are stable, we observe that the number of stable high-status leaders with high levels of *q* is equal to λ − 1. We also found that the number of relationships per individual has an impact on the transitions between phases. As that parameter increases, we observe how transitions between the three different types of leadership dynamics start to occur at lower values of *q* (see S1–S9 Figs).

### 3.3 Transient leader phase demonstrates a power vacuum

**Fig 4:**
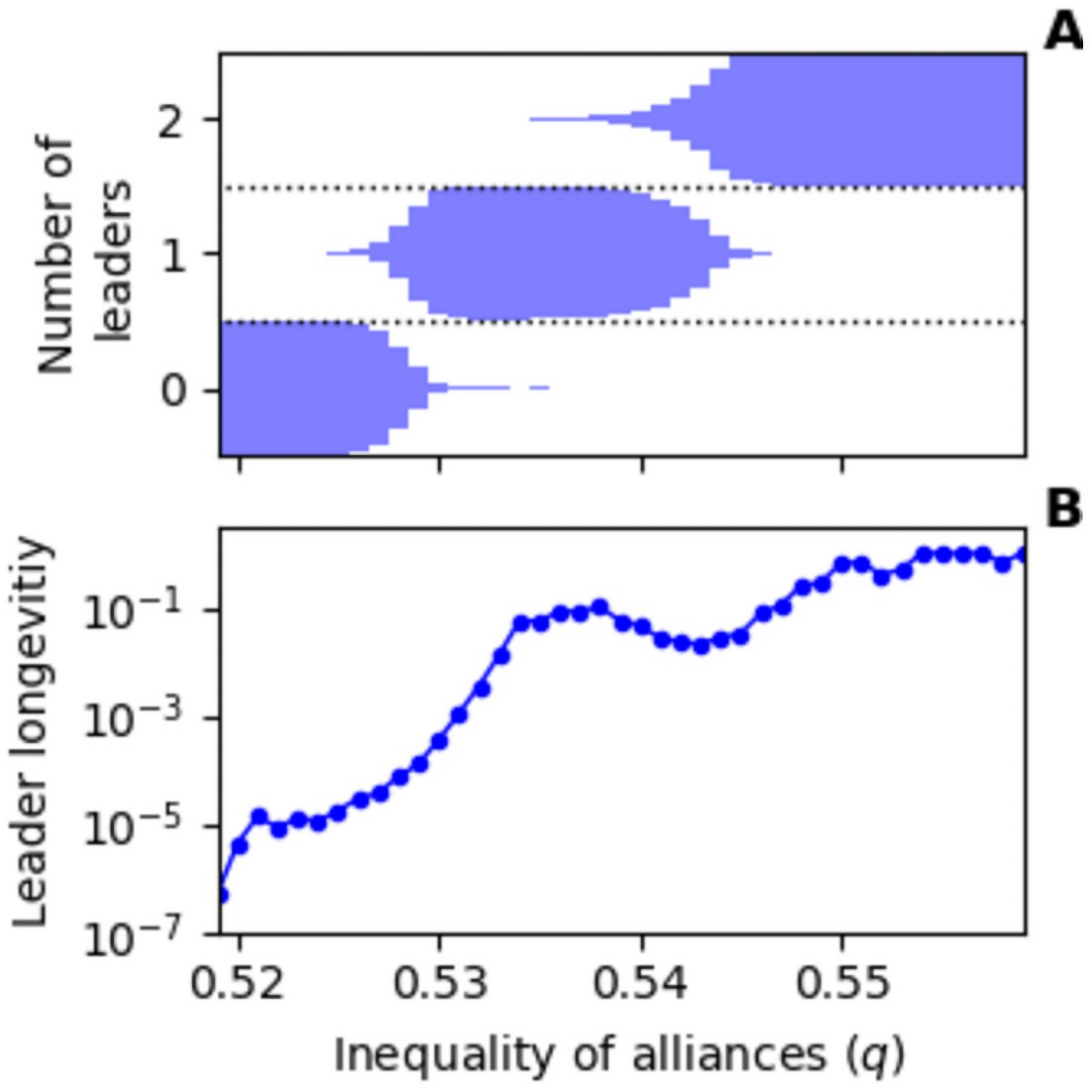
During transient-leader phases, as one leader loses status, another quickly replaces it. In panel **A**, the breadth of the horizontal bars represents the proportion of simulation timesteps with a specified number of leaders above a status threshold (*s*_*i*_ > 3.0). At *q* = 0.532 there was one leader over the threshold during 98% of simulation time steps. The average length of time, in terms of the proportion of the simulation, a leader stays above the threshold once they have reached the highest status level is shown in panel **B**. At *q* = 0.532, there is still a relatively high turnover of leaders with the average leader staying over the threshold for only a small fraction (0.0035) of the simulation. Unspecified parameters were as in Fig 2. Simulations were run for 2 million time steps.

An interesting phase in the dynamics demonstrates transient leaders (Fig 3, panel B). In this case, at any particular time-point in the simulation, there is only one high status leader. This leader can lose status in a riches-to-rags event, but another quickly replaces it. We have produced a video animation of the model of this phase of the model which is available in the S1 video. We ran simulations over a range of values for the inequality parameter (*q*) in Fig 4 which shows how there are ranges of the inequality parameter (*q*) where leader turnover is relatively high, but the number of leaders at any particular time-point is relatively constant, thus demonstrating a power-vacuum effect in our model.

### 3.4 Distribution of status and node degree

**Fig 5:**
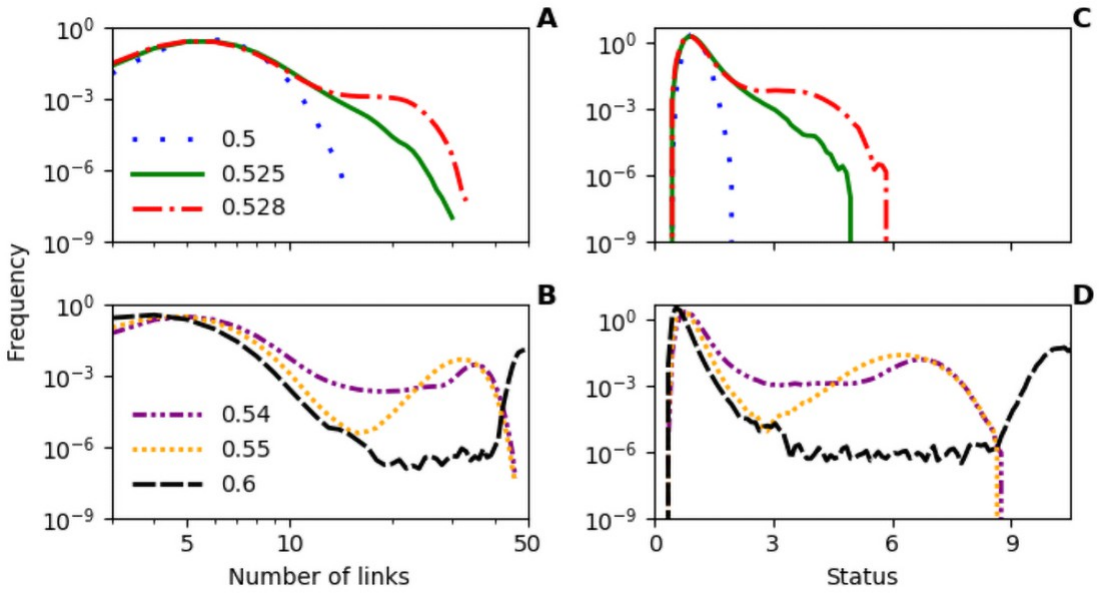
Frequency distributions of node degrees and status levels. As *q* is increased the distribution become increasing skewed. At higher values of *q*, the number of nodes becomes a factor with a second hump visible on the right-hand-side of both distributions. We can see how the rewiring of edges to an extra leader between *q* = 0.54 and *q* = 0.55 (see Fig 4 panel **A**) suppresses the frequencies of nodes with middling status or node-degree as *q* is increased. Parameters are the same as in Fig 3, *q* as shown. Simulations were run for 2 million time steps.

We find heavy-tailed distributions of node status and node degrees in our network model (Fig 5). We looked more closely at the distribution of node degrees for *q* = 0.525 using the Python Powerlaw package [26, 27]. As the parameters of our model stipulate that all nodes have at least degree 3, it makes sense to set a lower bound for the distribution we investigate, which we set to *X*_*min*_ = 6. A likelihood-ratio test [26] is used to compare the goodness of fit between the power-law distribution and two other distributions. We found no evidence (*p* ≪ 10^−100^) for either a log-normal or an exponential distribution compared with the power-law distribution (exponent of *P*(*x*) ∝ *x*^−8^). The relatively small range for the distribution is unusual for a power law and this is only found at a localised parameter value, but it does indicate that the distributions we find are unlikely to be explained by a simple log-normal or exponential distribution. Considering the distribution of node statuses, there is a cusp point of *s* ≈ 3.0 (Fig 5, panels C and D), where the frequency of nodes with *s* > 3.0 stops decreasing or starts to level off, which justifies our choice for using this as a threshold for defining leaders in Fig 4.

### 3.5 Shifts in leadership dynamics are consistent with the Neolithic

**Fig 6:**
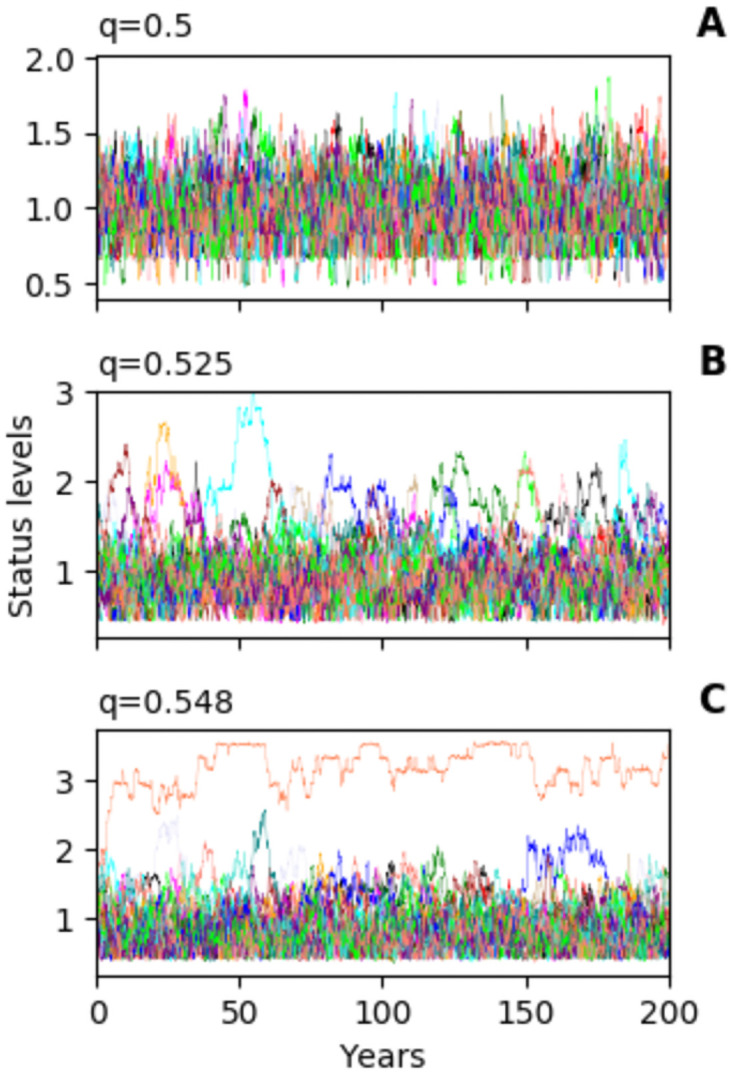
The model can demonstrate changes in leadership dynamics over time scales which correspond with those found over the Neolithic period. **A** No leader, **B** One leader which changes relatively frequently, **C** When leadership extends beyond a single lifespan, dynasties can emerge with status and leadership being passed to an inheritor. Parameters are the same as in Fig 2, with *w* = 0.01 and *r* = 0.05. One timestep represents one day.

In the introduction we argued that shifts in human leadership dynamics were due to technological advances that allowed individuals to control greater pools of resources. These advances would have had the effect of increasing the inequality parameter as seen in Fig 3. The analysis in that figure was done with a relatively high rewiring rate at the same frequency as status update. In human relationships, the rate at which relationships are changed is often at a relatively low frequency compared to how often status changes. For instance, new contracts take months to draw up but money and goods may change hands quite frequently. Adjusting the parameters, we can generate leadership dynamics over a broad range of timescales. We have selected one which is consistent with timelengths observed in the Neolithic era (see Fig 6).

## 4 Discussion

The model presented here demonstrates three different phases of leadership dynamics: a phase with no leader, a phase with changing leaders, and a phase with a constant leader or leaders. Which phase is present in the network depends on the inequality of relationships between individuals. This demonstrates how different leadership dynamics seen in human societies can be due to how status is transferred between the individual members of the society. This suggests that self-organisation of social norms around inequality can play a role in keeping a system parameter near to a critical point where leadership changes relatively frequently.

This work demonstrates a dynamic hierarchy in human networks where all individuals are have equivalent traits and fitness. Our model is a form of preferential-attachment, where nodes are more likely to connect to other nodes which are already of high status [22]. However, this is usually applied to growing networks [28, 29], and once one individual gains leadership, it is unlikely to change. In an alternative model, new individuals may dominate if they have a fitness advantage [30, 31]. Our model presents a *riches-to-rags* alternative where a high-status individual can lose status. In our model, we see how nodes have high numbers of connections (relationships) at some points and then other nodes take over. We find that the predicted exponent of our power law distribution is higher than that found in some friendship networks [22]. However friendship networks are only one type of relationship, and humans can relate to each other in many different ways. An example being where a chieftain controls access to food. Further study is needed to investigate how a model like ours can be challenged against empirical data.

Evidence for human societies with dynamic leaders during the Neolithic transitions [13] is consistent with the dynamic leader phase of our model. There is a transition between three phases of leadership dynamics in human societies from relatively egalitarian power structures, through a period where leaders change over time, to dominant institutionalised leaders [13]. Our model can be interpreted as a conceptual model for these leadership dynamics. Many have argued that control of surplus physical resources such as food and land, or intangible resources such as religious authority, can play an important role in promoting individuals to leadership rank [15, 19, 32, 33]. Having a surplus means an individual is able to form relationships where they need only exchange a small proportion of their resources, while their partners must exchange a larger proportion. Such inequality can be further exacerbated by scarcity of resources created by high population density [34]. This form of inequality is modelled by the level of the inequality parameter (*q*) in our model. Interestingly, our results present an alternative to this picture, suggesting that increases in the numbers of relationships per individual might also play an important role in creating conditions for absolutist power structures. More than one factor may have played a role in the transitions in leadership structure that happened during the Neolithic.

The three phases of human leadership dynamics correspond to three phases identified in the organisational psychology literature. Lewin has identified three modes of leadership: *Laissez Faire*, *Democratic* and *Autocratic* [35]. These three modes largely correspond to the three phases of leadership dynamics found in our model. Lewin’s study linked increasing control of central resources to more Democratic and Autocratic modes. A controlled surplus of this central resource enables a leader to pay off many individuals and maintain their leadership [36]. This reflects an inequality of alliances which is key to our model.

A interesting feature of our model is that it demonstrates heavy-tailed distributions of status and node-degree. Many systems are known to demonstrate such heavy-tailed distributions when they are at a critical point [37], i.e., when the rate-of-change of a variable is close zero. Further analysis of our model in the Supporting Information, which assumes that edge-rewiring is relatively slow compared to status update, shows an expected rate-of-change of node degree close to 0.0 when *q* ≈ 0.5. This suggests our model reaches a critical point, but further work is needed to investigate this in more detail.

The work we have presented has some limitations. The model we have presented is complex and difficult to analyse. Future models will hopefully simplify our approach while maintaining the interesting dynamics of changing leaders we have found in the model. Other models could add more realism, incorporating mortality of individuals and inheritance, or varying the numbers and types of relationship between individuals. Finally, it is important to find methods for challenging leadership models against data.

In this paper we focused primarily on applying this model to the development of insights regarding the Neolithic transitions from flat power structures to hierarchical societies. Future work can build upon these foundations to examine whether this model can be applied to other changes in societal structure, such as the movements from monarchy toward parliamentary democracies in 18th-century Europe, or a detailed study of the transitions of Roman civilization between various different structures including monarchy, through annually electing two concurrent consuls in the Roman Republic, a phase with three ‘Triumvirate’ leaders, to a single Imperator Caesar in the Roman Empire. As well as human societies, this theory can be of value to studying hierarchies in animal societies [38]. Other work might investigate the impact of relaxing some of our assumptions. For instance, exploring different rewiring rules where nodes have different numbers of edges, or rewire to others based on a similar or higher levels of status or numbers of edges. The model can also be extended in various ways to better represent the real-world contexts in which leadership dynamics operate; these could include representations of technological innovations, changes in social norms, or power struggles between potential leaders. These extensions would enable us to develop the model further into a powerful exploratory tool for human leadership dynamics.

## Supporting information

S1 File Mathematical analysis of the model. (PDF)

S1 Video Video showing the coevolutionary dynamic network evolving over time. A single individual is often at a high level of status compared to the others. Individuals with higher status are lighter coloured and have larger circles. Individuals are marked with ID numbers. The parameters are as in Panel B of Fig 2 in the main text.
(MP4)

S1 Fig Plot of the number of leaders over a variety of parameters of the model which was run over 5 million timesteps. At the top left of the figure, we see very fast turnover of leaders. As we increase *q*, leaders have increased time of leadership, at around 10^4^ the average leader has quite a long period with the highest status but there is still a large turnover. On the right side there are very few leaders in the chart and we see a single leader or several leaders. Parameters: *w* = 0.01, *n* = 20, and as shown in the figure.
(PNG)

S2 Fig Plot of the number of leaders over a variety of parameters of the model which was run over 5 million timesteps. At the top left of the figure, we see very fast turnover of leaders. As we increase *q*, leaders have increased time of leadership, at around 10^4^ the average leader has quite a long period with the highest status but there is still a large turnover. On the right side there are very few leaders in the chart and we see a single leader or several leaders. Parameters: *w* = 0.01, *n* = 100, and as shown in the figure.
(PNG)

S3 Fig Plot of the number of leaders over a variety of parameters of the model which was run over 5 million timesteps. At the top left of the figure, we see very fast turnover of leaders. As we increase *q*, leaders have increased time of leadership, at around 10^4^ the average leader has quite a long period with the highest status but there is still a large turnover. On the right side there are very few leaders in the chart and we see a single leader or several leaders. Parameters: *w* = 0.01, *n* = 1000, and as shown in the figure.
(PNG)

S4 Fig Plot of the number of leaders over a variety of parameters of the model which was run over 5 million timesteps. At the top left of the figure, we see very fast turnover of leaders. As we increase *q*, leaders have increased time of leadership, at around 10^4^ the average leader has quite a long period with the highest status but there is still a large turnover. On the right side there are very few leaders in the chart and we see a single leader or several leaders. Parameters: *w* = 0.1, *n* = 20, and as shown in the figure.
(PNG)

S5 Fig Plot of the number of leaders over a variety of parameters of the model which was run over 5 million timesteps. At the top left of the figure, we see very fast turnover of leaders. As we increase *q*, leaders have increased time of leadership, at around 10^4^ the average leader has quite a long period with the highest status but there is still a large turnover. On the right side there are very few leaders in the chart and we see a single leader or several leaders. Parameters: *w* = 0.1, *n* = 100, and as shown in the figure.
(PNG)

S6 Fig Plot of the number of leaders over a variety of parameters of the model which was run over 5 million timesteps. At the top left of the figure, we see very fast turnover of leaders. As we increase *q*, leaders have increased time of leadership, at around 10^4^ the average leader has quite a long period with the highest status but there is still a large turnover. On the right side there are very few leaders in the chart and we see a single leader or several leaders. Parameters: *w* = 0.1, *n* = 1000, and as shown in the figure.
(PNG)

S7 Fig Plot of the number of leaders over a variety of parameters of the model which was run over 5 million timesteps. At the top left of the figure, we see very fast turnover of leaders. As we increase *q*, leaders have increased time of leadership, at around 10^4^ the average leader has quite a long period with the highest status but there is still a large turnover. On the right side there are very few leaders in the chart and we see a single leader or several leaders. Parameters: *w* = 1.0, *n* = 20, and as shown in the figure.
(PNG)

S8 Fig Plot of the number of leaders over a variety of parameters of the model which was run over 5 million timesteps. At the top left of the figure, we see very fast turnover of leaders. As we increase *q*, leaders have increased time of leadership, at around 10^4^ the average leader has quite a long period with the highest status but there is still a large turnover. On the right side there are very few leaders in the chart and we see a single leader or several leaders. Parameters: *w* = 1.0, *n* = 100, and as shown in the figure.
(PNG)

S9 Fig Plot of the number of leaders over a variety of parameters of the model which was run over 5 million timesteps. At the top left of the figure, we see very fast turnover of leaders. As we increase *q*, leaders have increased time of leadership, at around 10^4^ the average leader has quite a long period with the highest status but there is still a large turnover. On the right side there are very few leaders in the chart and we see a single leader or several leaders. Parameters: *w* = 1.0, *n* = 1000, and as shown in the figure.
(PNG)
