# Supplementary material for: Modelling transitions between egalitarian, dynamic leader and absolutist power structures
Source: PLoS One. 2022 Feb 14;17(2):e0263665. doi: 10.1371/journal.pone.0263665 (PMC8843174; doi:10.1371/journal.pone.0263665)
Supplement: S1 File — (PDF) [file pone.0263665.s001.pdf]

Supporting Information: Modelling transitions between egalitarian, dynamic leader and absolutist power structures

John Bryden[1,2,*], Eric Silverman[3], Simon T. Powers[4]

1 School of Biological Sciences, Royal Holloway, University of London, Egham, TW20 0EX, United Kingdom
2 Observatory on Socia Media, Indiana University, Bloomington, Indiana, United States of America
3 MRC/CSO Social and Public Health Sciences Unit, University of Glasgow, Glasgow, United Kingdom
4 School of Computing, Edinburgh Napier University, Edinburgh, United Kingdom

* jabryden@iu.edu

1 Mathematical analysis of the model

1.1 Analysis of a single individual

We use a timescale-separation approach which assumes that status transmission between individuals will reach an equilibrium before rewiring takes place. For this to happen, this would need a relatively high level of r and a relatively low level of w. We focus on a single *leading* individual with a relatively high level of status s and a number of typical individuals x linking to it. After status transmission, if this individual contributes more status than an average individual, then it will maintain its number of links and potentially add new ones. We are able to calculate the contribution of the leader, relative to the average members, given the number of individuals linked to it and value of q and this is plotted in Fig. 1.

The figure shows how the analysis finds a critical point in the model at $q = 0.5$. When $q < 0.5$, the benefit to linking to an individual decreases as the number of links to that individual increases. When $q > 0.5$, the benefit to linking to an individual will increase as the number of links to that individual increases. When $q \approx 0.5$ there is little or no increase or decrease in benefit, and stochastic effects become increasingly important. At this point, a single individual may increase status to a higher level for a number of time steps before it then returns back to a similar level to the rest of the population.

1.2 Calculating the marginal link value

To analyse the model, we took the approach of looking at the case of a single focal individual. We introduce a timescale separation trick whereby we assume that the status of the focal individual (s_*) will reach an equilibrium value while the number of links to that individual is held constant at (x_*). Under this assumption, the identities of those linked to the focal individual may change, but the number will not. After the status of the individual has approached its equilibrium value, we can now compare the value of its links to those of a typical member of the population.

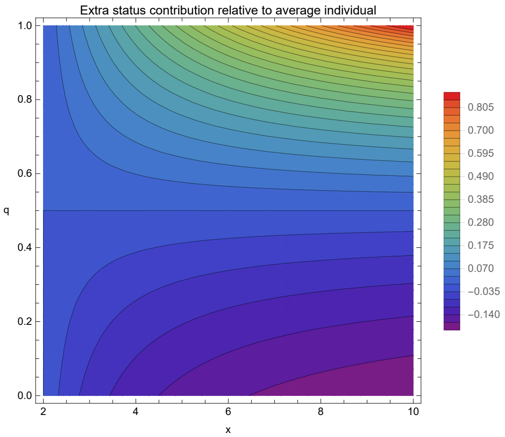

Fig S10. Plot showing the status contribution of an individual to any new link it receives, relative to the population-mean status contribution. This is plotted according to the number of links it has (x) and asymmetry in the model (q). In the horizontal blue region around $q = 0.5$, the model reaches a critical point where an individual will lose or gain links with equal probability, independent of x. Other parameters, $n = 100$, $\lambda = 2$.

The arithmetic mean status of non-focal member of the population (size n) is given by simply deducting the status of the focal individual from the total status and then dividing that by the number of non-focal members in the population.

$$\bar{s} = \frac{n - s_*}{n - 1} \ . \tag{1}$$

We consider the case where the focal-individual has x_* incoming links, and given each member of the population has λ outgoing links. The mean number of incoming links connected to non-focal population members is,

$$\bar{x} = \frac{\lambda n - x_*}{n - 1} \tag{2}$$

According to the rules of the model, each individual allocates a proportion r of its status to the value of all of its links. The focal individual contributes $rs_*/(x_* + \lambda)$ to each of its links and non-focal individuals contribute $r\bar{s}/(\bar{x} + \lambda)$ to each of their links. The focal individual will receive a proportion q of the value of each of its incoming links and a proportion $1 - q$ of the value of outgoing links. The change in status of the focal individual is given by

$$\delta \bar{s_*} = (x_* q + \lambda(1 - q))\, r\bar{s}/(\bar{x} + \lambda) - rs_* \tag{3}$$

To find an equilibrium we can substitute $\delta s_* = 0$ and solve Eq. (3) for s_*. This gives

$$\tilde{s_*} = \frac{n(\lambda + x_*)\,(qx_* - \lambda(q - 1))}{\lambda^2(2(n - 1)q + 1) - 2\lambda n(q - 1)x_* + (2q - 1)x_*^2} \ .$$

To understand whether the focal individual will increase in status and links or not, we need to look at the marginal value of links to that individual compared with links to average members of the population. We assume that the focal individual has reached the equilibrium point ($s_e = \tilde{s_*}$ without changing the number of links x_*. At this point, the marginal status value of linking to the focal individual is given by,

$$\hat{s_*} = r\frac{s_*}{x_* + \lambda} - r\frac{\bar{s}}{\bar{x} + \lambda}$$

We can substitute $s_e = \tilde{s_*}$ into this equation to give,

$$\hat{s} = \frac{n(2q-1)(x_* - \lambda)}{\lambda^2(2(n-1)q+1) - 2\lambda n(q-1)x_* + (2q-1)x_*^2} \,. \tag{4}$$

This equation will give us the marginal link value $\hat{s}$ for links to a focal individual compared to links to a typical individual. When this is close to zero, the focal individual can lose or gain links with equal probability. When this number is less than zero, the focal individual will tend to lose links. When this number is greater than zero, the focal individual will tend to gain links.
