# Supplementary figures and images for: Modelling transitions between egalitarian, dynamic leader and absolutist power structures
Source: PLoS One. 2022 Feb 14;17(2):e0263665. doi: 10.1371/journal.pone.0263665 (PMC8843174; doi:10.1371/journal.pone.0263665)

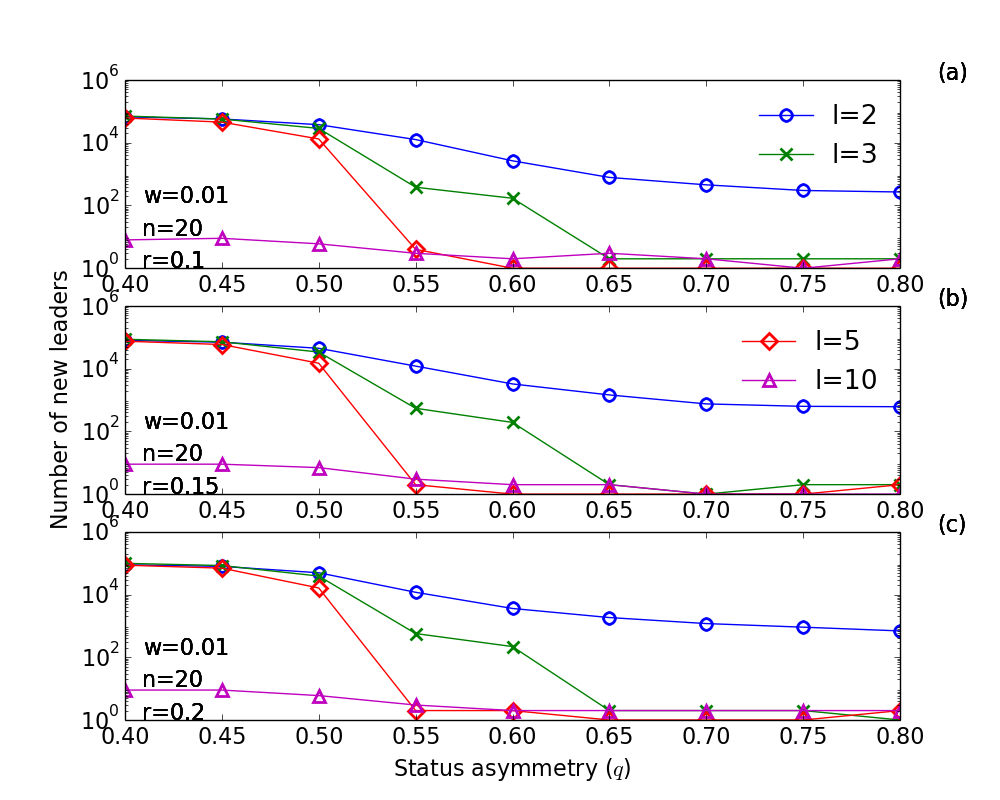

Supplement: S1 Fig — At the top left of the figure, we see very fast turnover of leaders. As we increase q, leaders have increased time of leadership, at around 104 the average leader has quite a long period with the highest status but there is still a large turnover. On the right side there are very few leaders in the chart and we see a single leader or several leaders. Parameters: w = 0.01, n = 20, and as shown in the figure. (PNG) [file pone.0263665.s003.png]

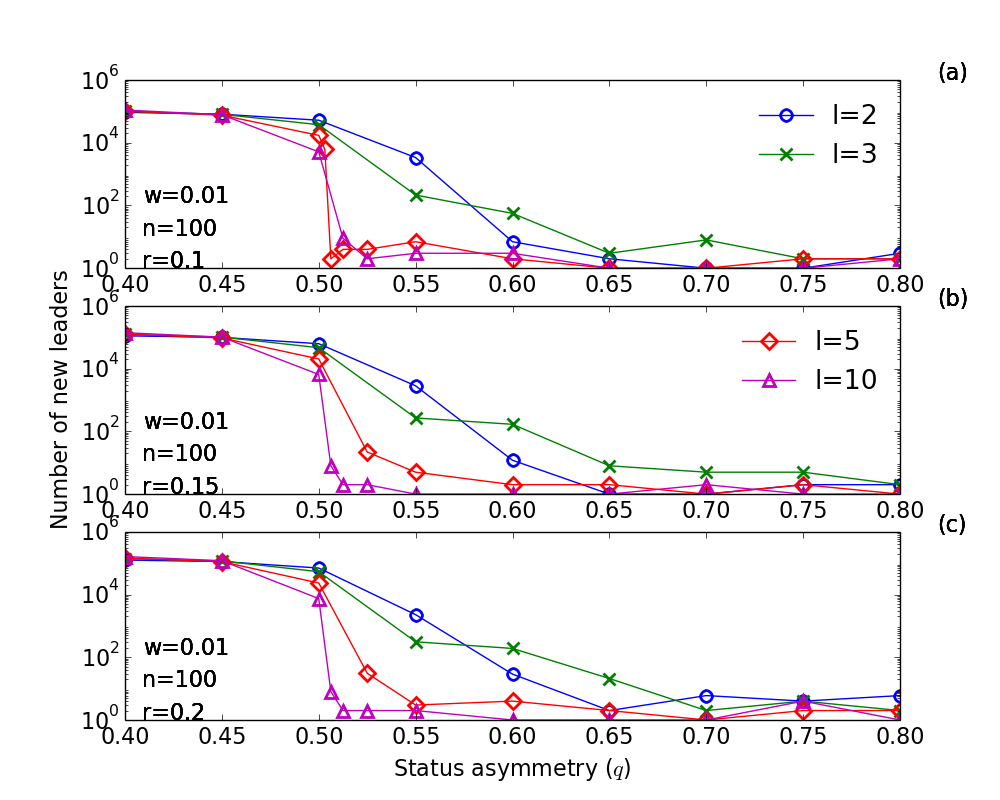

Supplement: S2 Fig — At the top left of the figure, we see very fast turnover of leaders. As we increase q, leaders have increased time of leadership, at around 104 the average leader has quite a long period with the highest status but there is still a large turnover. On the right side there are very few leaders in the chart and we see a single leader or several leaders. Parameters: w = 0.01, n = 100, and as shown in the figure. (PNG) [file pone.0263665.s004.png]

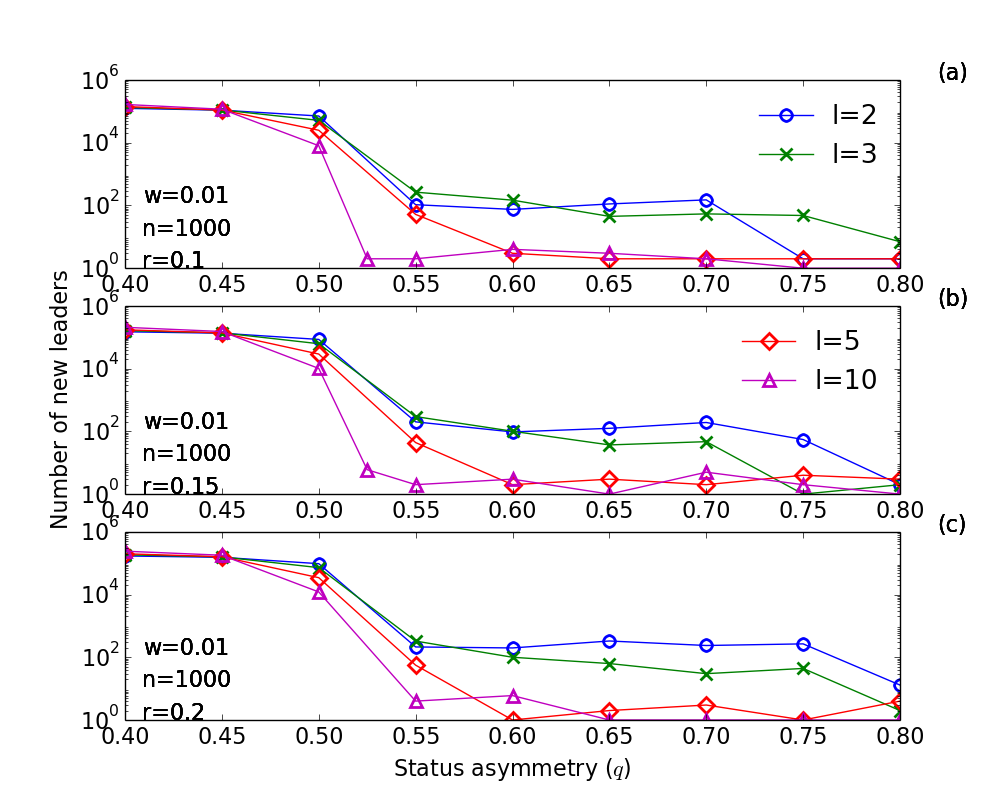

Supplement: S3 Fig — At the top left of the figure, we see very fast turnover of leaders. As we increase q, leaders have increased time of leadership, at around 104 the average leader has quite a long period with the highest status but there is still a large turnover. On the right side there are very few leaders in the chart and we see a single leader or several leaders. Parameters: w = 0.01, n = 1000, and as shown in the figure. (PNG) [file pone.0263665.s005.png]

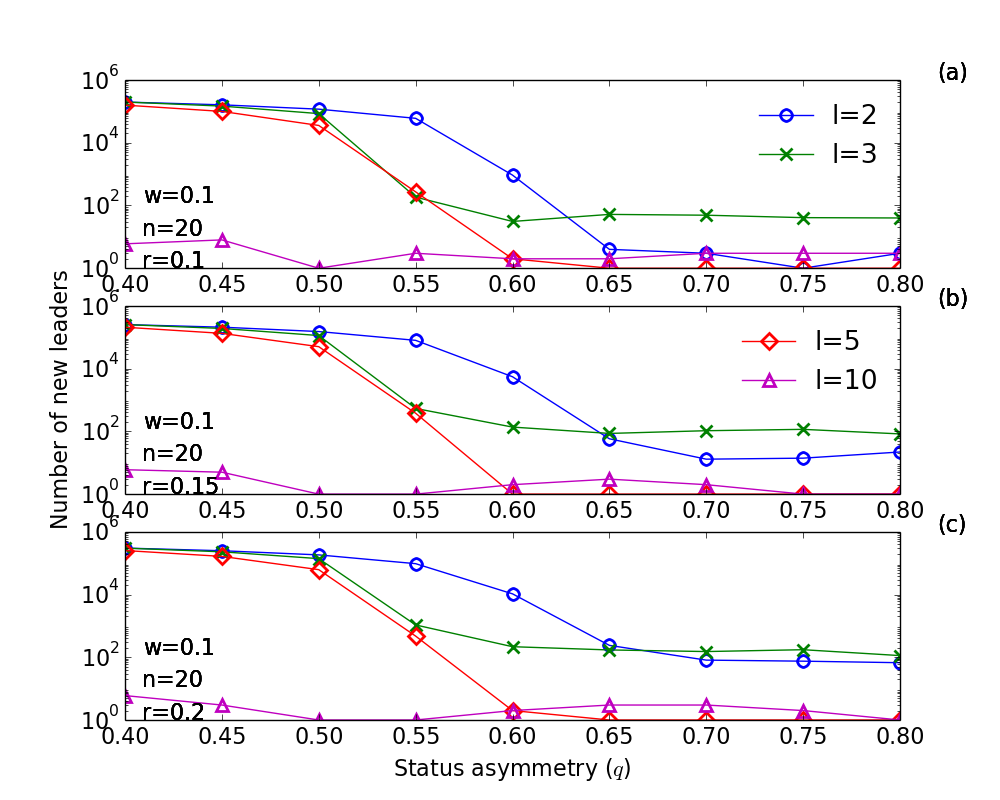

Supplement: S4 Fig — At the top left of the figure, we see very fast turnover of leaders. As we increase q, leaders have increased time of leadership, at around 104 the average leader has quite a long period with the highest status but there is still a large turnover. On the right side there are very few leaders in the chart and we see a single leader or several leaders. Parameters: w = 0.1, n = 20, and as shown in the figure. (PNG) [file pone.0263665.s006.png]

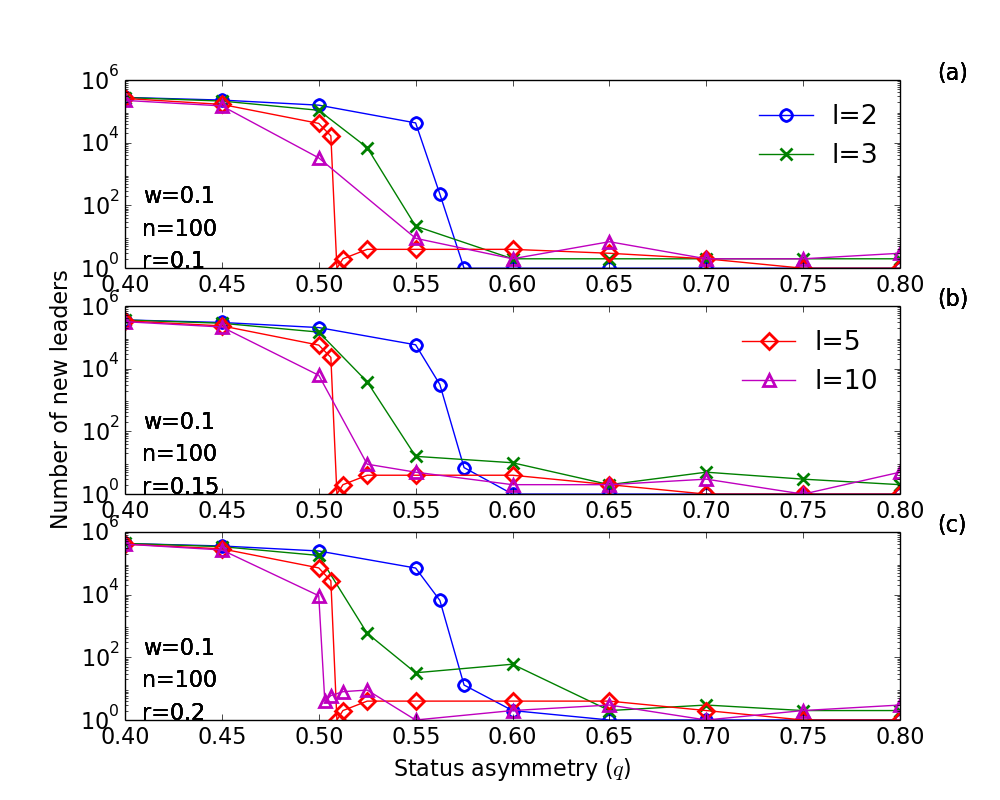

Supplement: S5 Fig — At the top left of the figure, we see very fast turnover of leaders. As we increase q, leaders have increased time of leadership, at around 104 the average leader has quite a long period with the highest status but there is still a large turnover. On the right side there are very few leaders in the chart and we see a single leader or several leaders. Parameters: w = 0.1, n = 100, and as shown in the figure. (PNG) [file pone.0263665.s007.png]

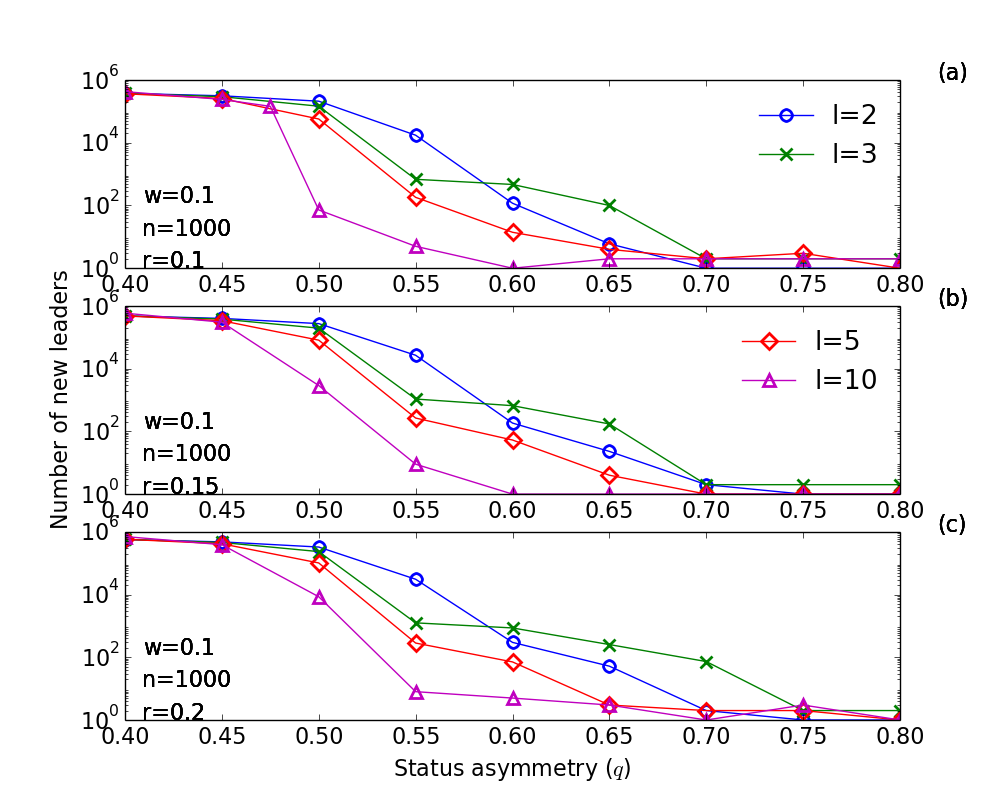

Supplement: S6 Fig — At the top left of the figure, we see very fast turnover of leaders. As we increase q, leaders have increased time of leadership, at around 104 the average leader has quite a long period with the highest status but there is still a large turnover. On the right side there are very few leaders in the chart and we see a single leader or several leaders. Parameters: w = 0.1, n = 1000, and as shown in the figure. (PNG) [file pone.0263665.s008.png]

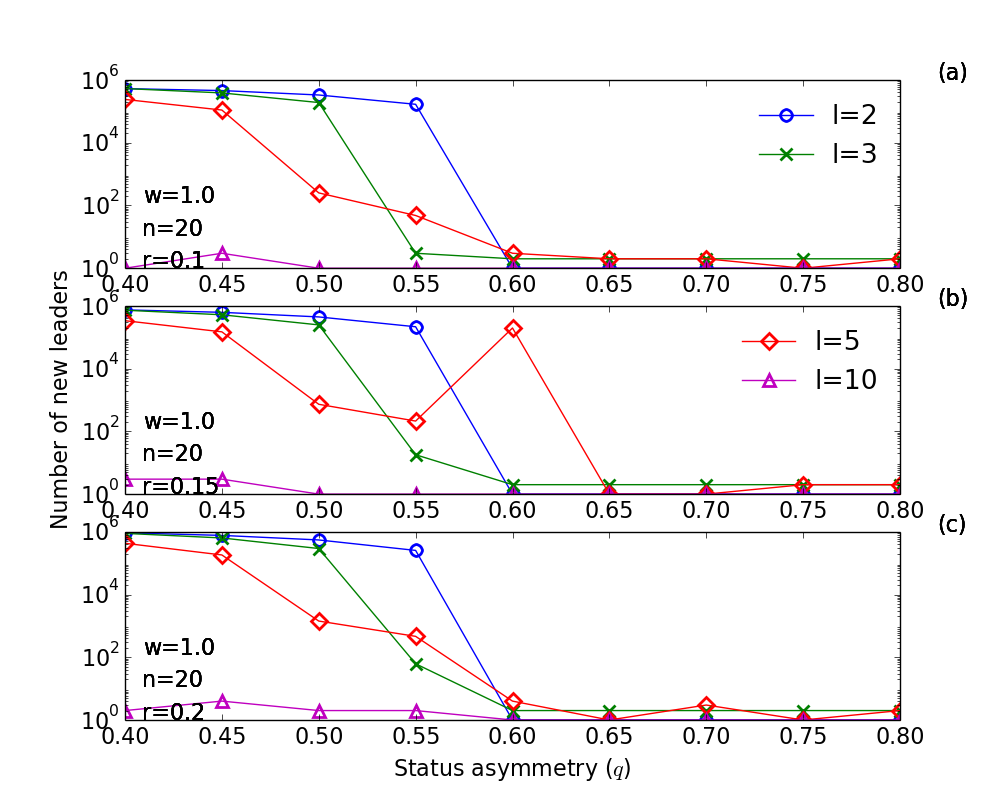

Supplement: S7 Fig — At the top left of the figure, we see very fast turnover of leaders. As we increase q, leaders have increased time of leadership, at around 104 the average leader has quite a long period with the highest status but there is still a large turnover. On the right side there are very few leaders in the chart and we see a single leader or several leaders. Parameters: w = 1.0, n = 20, and as shown in the figure. (PNG) [file pone.0263665.s009.png]

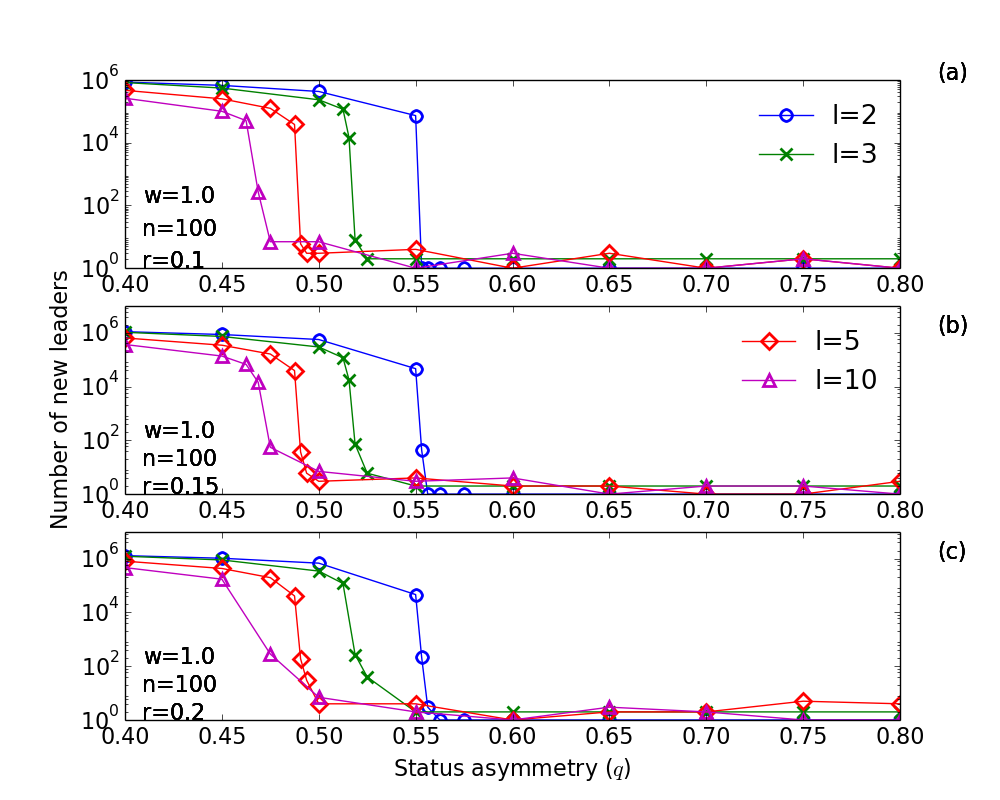

Supplement: S8 Fig — At the top left of the figure, we see very fast turnover of leaders. As we increase q, leaders have increased time of leadership, at around 104 the average leader has quite a long period with the highest status but there is still a large turnover. On the right side there are very few leaders in the chart and we see a single leader or several leaders. Parameters: w = 1.0, n = 100, and as shown in the figure. (PNG) [file pone.0263665.s010.png]

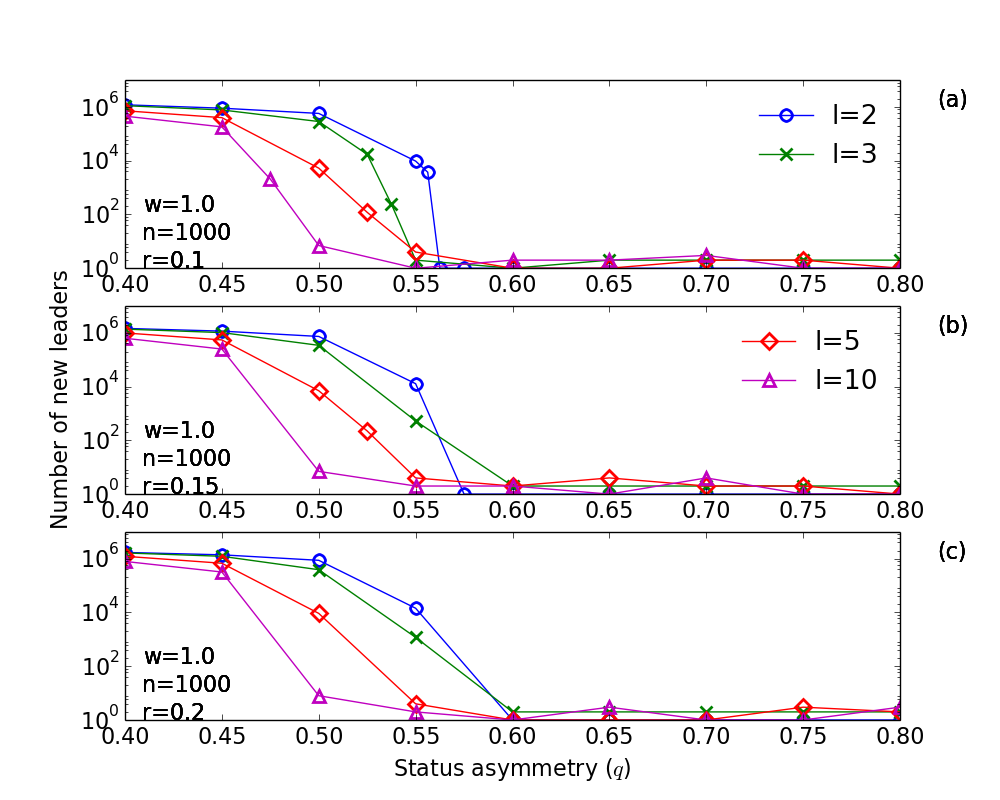

Supplement: S9 Fig — At the top left of the figure, we see very fast turnover of leaders. As we increase q, leaders have increased time of leadership, at around 104 the average leader has quite a long period with the highest status but there is still a large turnover. On the right side there are very few leaders in the chart and we see a single leader or several leaders. Parameters: w = 1.0, n = 1000, and as shown in the figure. (PNG) [file pone.0263665.s011.png]
